# (5,7,7,12,14,14-Hexamethyl-1,4,8,11-tetra­azacyclo­tetra­deca-4,11-diene-κ^4^ *N* ^1^,*N* ^4^,*N* ^8^,*N* ^11^)(thio­cyanato-κ*S*)nickel(II) perchlorate monohydrate

**DOI:** 10.1107/S1600536809018091

**Published:** 2009-05-20

**Authors:** Jian-Hong Bi

**Affiliations:** a Deparment of Chemistry and Chemical Engineering, Hefei Teachers College, Hefei 230061, People’s Republic of China

## Abstract

In the title compound, [Ni(SCN)(C_16_H_32_N_4_)]ClO_4_·H_2_O, the Ni^II^ ion is coordinated by the four N atoms of the tetra­azacyclo­tetra­deca-4,11-diene macrocyclic ligand and by the S atom of a thio­cyanate anion. The perchlorate anion is rotationally disordered around one Cl—O bond between two orientations; the occupancies refined to 0.61 (4) and 0.39 (4). Inter­molecular O—H⋯N, N—H⋯O and N—H⋯N hydrogen bonds link two cations, two anions and two solvent water mol­ecules into a centrosymmetric cluster. The crystal packing is further stabilized by weak inter­molecular C—H⋯O hydrogen bonds.

## Related literature

For the crystal structures of related complexes, see: Bienko *et al.* (2007 ▶); Shen *et al.* (1999 ▶); Szalda & Fujita (1992 ▶).
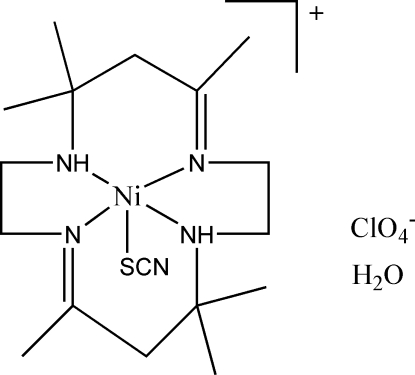


## Experimental

### 

#### Crystal data

[Ni(NCS)(C_16_H_32_N_4_)]ClO_4_·H_2_O
*M* *_r_* = 514.71
Triclinic, 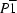

*a* = 7.2678 (11) Å
*b* = 8.9998 (13) Å
*c* = 19.513 (2) Å
α = 84.1430 (10)°
β = 87.005 (2)°
γ = 67.3480 (10)°
*V* = 1171.6 (3) Å^3^
*Z* = 2
Mo *K*α radiation
μ = 1.07 mm^−1^
*T* = 291 K
0.49 × 0.40 × 0.39 mm

#### Data collection

Bruker SMART CCD area-detector diffractometer
Absorption correction: multi-scan (*SADABS*; Bruker, 2000 ▶) *T*_min_ = 0.623, *T*_max_ = 0.681
6103 measured reflections
4062 independent reflections
3247 reflections with *I* > 2σ(*I*)
*R*_int_ = 0.019

#### Refinement

*R*[*F* ^2^ > 2σ(*F* ^2^)] = 0.041
*wR*(*F* ^2^) = 0.109
*S* = 1.03
4062 reflections
299 parameters
H-atom parameters constrained
Δρ_max_ = 0.53 e Å^−3^
Δρ_min_ = −0.90 e Å^−3^

### 

Data collection: *SMART* (Bruker, 2000 ▶); cell refinement: *SAINT* (Bruker, 2000 ▶); data reduction: *SAINT*; program(s) used to solve structure: *SHELXTL* (Sheldrick, 2008 ▶); program(s) used to refine structure: *SHELXTL*; molecular graphics: *SHELXTL*; software used to prepare material for publication: *SHELXTL*.

## Supplementary Material

Crystal structure: contains datablocks I, global. DOI: 10.1107/S1600536809018091/cv2562sup1.cif

Structure factors: contains datablocks I. DOI: 10.1107/S1600536809018091/cv2562Isup2.hkl

Additional supplementary materials:  crystallographic information; 3D view; checkCIF report

## Figures and Tables

**Table 1:** Hydrogen-bond geometry (Å, °)

*D*—H⋯*A*	*D*—H	H⋯*A*	*D*⋯*A*	*D*—H⋯*A*
N2—H2⋯O2	0.91	2.28	3.16 (3)	162
N4—H4⋯N5	0.91	2.33	3.241 (5)	175
O5—H5*F*⋯N5	0.85	2.09	2.942 (6)	178
O5—H5*G*⋯N5^i^	0.85	2.15	2.997 (6)	178
C3—H3*A*⋯O4^ii^	0.97	2.49	3.450 (16)	172
C3—H3*B*⋯O2	0.97	2.48	3.26 (3)	137
C15—H15*A*⋯O1^iii^	0.97	2.37	3.155 (6)	138

Symmetry codes: (i) 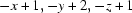; (ii) 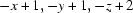; (iii) 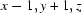.

**Fig. 1.**
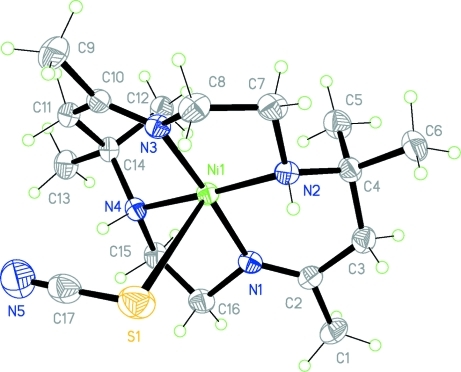
Molecular structure of the cation of the title compound showing 30% probability displacement ellipsoids and the atomic numbering.

## supplementary crystallographic information

### Comment

A number of researches study azamacrocyclic systems (Bienko *et al.*, 2007; Shen *et al.*, 1999; Szalda *et al.*, 1992). Szalda reported a crystal structure of metal complex derived from tetraazacyclotetradeca-4,11-diene macrocycles (Szalda *et al.*, 1992). To investigate whether the potentially explosive perchlorate anions in this complex can be replaced by other anions to facilitate its further application, NCS^-^ anion was used and the title complex was obtained.

The coordination geometry of Ni^II^ center is shown in Fig.1. The Ni^II^ center adopts a square-pyramidal coordination geometry, where four N atoms from macrocyclic ligand form an equatorial plane and one S atom from the thiocyanate anion occupies an apical position. The Ni–S bond length of 3.298 (13) Å is slightly longer than those of 3.171 (14) Å observed and discussed by Bienko *et al.* (2007).

The crystal packing is stabilized by intermolecular hydrogen bonding interactions (Table 1).

### Experimental

All solvents and chemicals were of analytical grade and were used without further purification. The mononuclear nickel(II)-diperchlorate macrocycle complex (0.538 g, 0.1 mmol), which was prepared *via* similar method as reported previously (Szalda *et al.*, 1992), was dissolved in acetonitrile (30 ml) and NH_4_(NCS)(0.152 g, 0.2 mmol) was added. The mixture was refluxed for 2 h, and then cooled to room temperature. The green precipitate was collected, washed with a small amount of acetonitrile and dried *in vacuo*. Single crystals suitable for X-ray analysis were grown from the mother solution by slow evaporation at room temperature in air. Elemental analysis calculated for C_17_H_34_ClN_5_NiO_5_S: C 39.67, H 6.66, N 13.61%; found: C 39.71, H 6.70, N 13.57%.

### Refinement

All hydrogen atoms were geometrically positioned (C—H 0.93–0.97 Å, O–H 0.84–0.85 Å, N–H 0.91 Å) and refined as riding, with *U*_iso_(H)=1.2–1.5 *U*_eq_ of the parent atom. The oxygen atoms O2, O3 and O4 of the perchlorate anion were treated as disordered between two orientions with the occupancies refined to 0.61 (4) and 0.39 (4).

### Crystal data

**Table:**

[Ni(NCS)(C_16_H_32_N_4_)]ClO_4_·H_2_O	*Z* = 2
*M**_r_* = 514.71	*F*(000) = 544
Triclinic, *P*1	*D*_x_ = 1.459 Mg m^−^^3^
Hall symbol: -P 1	Mo *K*α radiation, λ = 0.71073 Å
*a* = 7.2678 (11) Å	Cell parameters from 2923 reflections
*b* = 8.9998 (13) Å	θ = 2.5–27.7°
*c* = 19.513 (2) Å	µ = 1.07 mm^−^^1^
α = 84.143 (1)°	*T* = 291 K
β = 87.005 (2)°	Block, green
γ = 67.348 (1)°	0.49 × 0.40 × 0.39 mm
*V* = 1171.6 (3) Å^3^	

### Data collection

**Table:**

Bruker SMART CCD area-detector diffractometer	4062 independent reflections
Radiation source: fine-focus sealed tube	3247 reflections with *I* > 2σ(*I*)
graphite	*R*_int_ = 0.019
φ and ω scans	θ_max_ = 25.0°, θ_min_ = 2.1°
Absorption correction: multi-scan (*SADABS*; Bruker, 2000)	*h* = −8→8
*T*_min_ = 0.623, *T*_max_ = 0.681	*k* = −10→8
6103 measured reflections	*l* = −23→16

### Refinement

**Table:**

Refinement on *F*^2^	Primary atom site location: structure-invariant direct methods
Least-squares matrix: full	Secondary atom site location: difference Fourier map
*R*[*F*^2^ > 2σ(*F*^2^)] = 0.041	Hydrogen site location: inferred from neighbouring sites
*wR*(*F*^2^) = 0.109	H-atom parameters constrained
*S* = 1.03	*w* = 1/[σ^2^(*F*_o_^2^) + (0.0501*P*)^2^ + 0.9082*P*] where *P* = (*F*_o_^2^ + 2*F*_c_^2^)/3
4062 reflections	(Δ/σ)_max_ = 0.001
299 parameters	Δρ_max_ = 0.53 e Å^−^^3^
0 restraints	Δρ_min_ = −0.89 e Å^−^^3^

### Special details

**Table:**

Experimental. The structure was solved by direct methods (Bruker, 2000) and successive difference Fourier syntheses.
Geometry. All e.s.d.'s (except the e.s.d. in the dihedral angle between two l.s. planes) are estimated using the full covariance matrix. The cell e.s.d.'s are taken into account individually in the estimation of e.s.d.'s in distances, angles and torsion angles; correlations between e.s.d.'s in cell parameters are only used when they are defined by crystal symmetry. An approximate (isotropic) treatment of cell e.s.d.'s is used for estimating e.s.d.'s involving l.s. planes.
Refinement. Refinement of *F*^2^ against ALL reflections. The weighted *R*-factor *wR* and goodness of fit *S* are based on *F*^2^, conventional *R*-factors *R* are based on *F*, with *F* set to zero for negative *F*^2^. The threshold expression of *F*^2^ > σ(*F*^2^) is used only for calculating *R*-factors(gt) *etc*. and is not relevant to the choice of reflections for refinement. *R*-factors based on *F*^2^ are statistically about twice as large as those based on *F*, and *R*- factors based on ALL data will be even larger.

### Fractional atomic coordinates and isotropic or equivalent isotropic displacement parameters (Å^2^)

**Table:**

	*x*	*y*	*z*	*U*_iso_*/*U*_eq_	Occ. (<1)
Ni1	0.30773 (6)	0.73405 (4)	0.74822 (2)	0.03298 (14)	
S1	0.77119 (17)	0.55310 (15)	0.69389 (7)	0.0742 (3)	
Cl1	0.83588 (13)	0.23413 (11)	0.87389 (5)	0.0488 (2)	
N1	0.3810 (4)	0.8445 (3)	0.81175 (14)	0.0369 (6)	
N2	0.3265 (4)	0.5584 (3)	0.81554 (13)	0.0358 (6)	
H2	0.4585	0.5040	0.8246	0.043*	
N3	0.2600 (4)	0.6153 (3)	0.68168 (14)	0.0370 (6)	
N4	0.2731 (4)	0.9145 (3)	0.68173 (13)	0.0358 (6)	
H4	0.3769	0.8812	0.6512	0.043*	
N5	0.6503 (6)	0.7714 (6)	0.5780 (2)	0.0895 (13)	
O1	1.0226 (5)	0.1857 (5)	0.8446 (3)	0.1262 (16)	
O2	0.758 (4)	0.401 (3)	0.8792 (15)	0.103 (6)	0.61 (4)
O3	0.698 (3)	0.198 (3)	0.8398 (14)	0.115 (7)	0.61 (4)
O4	0.889 (5)	0.1340 (19)	0.9359 (6)	0.151 (8)	0.61 (4)
O2'	0.765 (6)	0.150 (3)	0.9228 (18)	0.125 (11)	0.39 (4)
O3'	0.724 (6)	0.244 (5)	0.8153 (16)	0.132 (12)	0.39 (4)
O4'	0.774 (7)	0.381 (6)	0.9037 (19)	0.101 (10)	0.39 (4)
O5	0.4522 (5)	0.8714 (5)	0.44382 (19)	0.1003 (11)	
H5F	0.5099	0.8396	0.4824	0.120*	
H5G	0.4217	0.9727	0.4365	0.120*	
C1	0.4568 (6)	0.9196 (5)	0.9214 (2)	0.0567 (10)	
H1A	0.5602	0.9470	0.8977	0.085*	
H1B	0.5066	0.8575	0.9642	0.085*	
H1C	0.3455	1.0168	0.9303	0.085*	
C2	0.3917 (5)	0.8227 (4)	0.87771 (17)	0.0393 (7)	
C3	0.3413 (5)	0.6913 (4)	0.91705 (18)	0.0470 (8)	
H3A	0.2667	0.7355	0.9579	0.056*	
H3B	0.4658	0.6070	0.9327	0.056*	
C4	0.2248 (5)	0.6103 (4)	0.88317 (17)	0.0423 (8)	
C5	0.0098 (5)	0.7264 (5)	0.8714 (2)	0.0587 (10)	
H5A	0.0078	0.8222	0.8442	0.088*	
H5B	−0.0551	0.7550	0.9150	0.088*	
H5C	−0.0589	0.6753	0.8476	0.088*	
C6	0.2333 (6)	0.4619 (5)	0.9316 (2)	0.0595 (10)	
H6A	0.1473	0.4156	0.9148	0.089*	
H6B	0.1902	0.4942	0.9770	0.089*	
H6C	0.3676	0.3832	0.9332	0.089*	
C7	0.2598 (6)	0.4456 (4)	0.78297 (19)	0.0482 (9)	
H7A	0.1158	0.4814	0.7863	0.058*	
H7B	0.3180	0.3379	0.8063	0.058*	
C8	0.3252 (6)	0.4434 (4)	0.70907 (19)	0.0493 (9)	
H8A	0.4689	0.3895	0.7052	0.059*	
H8B	0.2635	0.3874	0.6840	0.059*	
C9	0.2099 (7)	0.5436 (5)	0.5678 (2)	0.0617 (11)	
H9A	0.3387	0.4569	0.5687	0.093*	
H9B	0.1862	0.5996	0.5226	0.093*	
H9C	0.1092	0.5004	0.5794	0.093*	
C10	0.2029 (5)	0.6592 (4)	0.61938 (17)	0.0404 (8)	
C11	0.1245 (5)	0.8328 (4)	0.59141 (17)	0.0432 (8)	
H11A	0.0002	0.8546	0.5685	0.052*	
H11B	0.2179	0.8455	0.5562	0.052*	
C12	0.0857 (5)	0.9639 (4)	0.64053 (16)	0.0394 (7)	
C13	−0.0909 (5)	0.9766 (4)	0.68875 (18)	0.0466 (8)	
H13A	−0.0751	0.8710	0.7093	0.070*	
H13B	−0.2121	1.0222	0.6631	0.070*	
H13C	−0.0965	1.0448	0.7242	0.070*	
C14	0.0433 (6)	1.1253 (4)	0.5974 (2)	0.0554 (10)	
H14A	0.0124	1.2101	0.6275	0.083*	
H14B	−0.0678	1.1476	0.5680	0.083*	
H14C	0.1587	1.1194	0.5698	0.083*	
C15	0.2952 (6)	1.0464 (4)	0.71649 (18)	0.0473 (9)	
H15A	0.1677	1.1145	0.7353	0.057*	
H15B	0.3427	1.1128	0.6838	0.057*	
C16	0.4423 (6)	0.9688 (4)	0.77314 (19)	0.0487 (9)	
H16A	0.5759	0.9194	0.7542	0.058*	
H16B	0.4409	1.0486	0.8032	0.058*	
C17	0.7045 (6)	0.6772 (5)	0.6252 (3)	0.0621 (11)	

### Atomic displacement parameters (Å^2^)

**Table:**

	*U*^11^	*U*^22^	*U*^33^	*U*^12^	*U*^13^	*U*^23^
Ni1	0.0374 (2)	0.0284 (2)	0.0350 (2)	−0.01414 (17)	0.00074 (17)	−0.00531 (16)
S1	0.0542 (6)	0.0768 (8)	0.0868 (9)	−0.0221 (6)	−0.0052 (6)	0.0040 (6)
Cl1	0.0432 (5)	0.0483 (5)	0.0450 (5)	−0.0061 (4)	0.0094 (4)	−0.0117 (4)
N1	0.0370 (14)	0.0317 (14)	0.0435 (16)	−0.0140 (12)	0.0022 (12)	−0.0078 (12)
N2	0.0340 (14)	0.0309 (14)	0.0409 (15)	−0.0111 (11)	−0.0024 (11)	−0.0012 (11)
N3	0.0396 (15)	0.0305 (14)	0.0428 (16)	−0.0149 (12)	0.0008 (12)	−0.0070 (12)
N4	0.0425 (15)	0.0315 (14)	0.0356 (15)	−0.0164 (12)	0.0068 (12)	−0.0078 (11)
N5	0.071 (3)	0.098 (3)	0.082 (3)	−0.017 (2)	0.000 (2)	0.007 (3)
O1	0.070 (2)	0.144 (4)	0.160 (4)	−0.029 (2)	0.041 (2)	−0.062 (3)
O2	0.083 (7)	0.074 (6)	0.143 (16)	−0.012 (5)	−0.027 (11)	−0.026 (10)
O3	0.078 (5)	0.119 (9)	0.169 (19)	−0.053 (6)	−0.008 (9)	−0.040 (11)
O4	0.158 (16)	0.151 (8)	0.071 (5)	0.013 (9)	−0.002 (7)	0.027 (5)
O2'	0.13 (2)	0.110 (13)	0.125 (17)	−0.050 (13)	0.048 (15)	−0.002 (11)
O3'	0.121 (19)	0.15 (2)	0.081 (13)	0.002 (13)	−0.035 (11)	−0.040 (12)
O4'	0.10 (2)	0.10 (2)	0.104 (17)	−0.026 (15)	0.031 (14)	−0.071 (17)
O5	0.106 (3)	0.114 (3)	0.085 (3)	−0.045 (2)	−0.019 (2)	−0.004 (2)
C1	0.063 (2)	0.063 (2)	0.049 (2)	−0.026 (2)	−0.0088 (19)	−0.0173 (19)
C2	0.0320 (16)	0.0397 (18)	0.0408 (19)	−0.0060 (14)	0.0001 (14)	−0.0115 (15)
C3	0.051 (2)	0.052 (2)	0.0383 (19)	−0.0191 (17)	0.0002 (16)	−0.0057 (16)
C4	0.0389 (18)	0.0453 (19)	0.0405 (19)	−0.0151 (15)	0.0031 (15)	0.0002 (15)
C5	0.042 (2)	0.065 (3)	0.061 (3)	−0.0130 (18)	0.0071 (18)	−0.003 (2)
C6	0.063 (2)	0.065 (3)	0.053 (2)	−0.032 (2)	0.0009 (19)	0.0119 (19)
C7	0.058 (2)	0.0326 (18)	0.058 (2)	−0.0216 (17)	−0.0078 (18)	0.0007 (16)
C8	0.063 (2)	0.0290 (17)	0.058 (2)	−0.0171 (16)	−0.0087 (18)	−0.0095 (16)
C9	0.080 (3)	0.054 (2)	0.054 (2)	−0.024 (2)	−0.001 (2)	−0.0235 (19)
C10	0.0436 (19)	0.0434 (19)	0.0402 (19)	−0.0219 (16)	0.0069 (15)	−0.0135 (15)
C11	0.0467 (19)	0.049 (2)	0.0362 (18)	−0.0204 (17)	0.0019 (15)	−0.0068 (15)
C12	0.0427 (18)	0.0382 (18)	0.0333 (17)	−0.0118 (15)	0.0022 (14)	−0.0020 (14)
C13	0.0398 (18)	0.049 (2)	0.044 (2)	−0.0088 (16)	0.0031 (15)	−0.0065 (16)
C14	0.066 (2)	0.044 (2)	0.049 (2)	−0.0161 (19)	−0.0030 (19)	0.0058 (17)
C15	0.067 (2)	0.0337 (18)	0.047 (2)	−0.0252 (17)	0.0034 (17)	−0.0061 (15)
C16	0.061 (2)	0.045 (2)	0.053 (2)	−0.0321 (18)	0.0022 (18)	−0.0107 (17)
C17	0.041 (2)	0.066 (3)	0.075 (3)	−0.015 (2)	0.006 (2)	−0.016 (2)

### Geometric parameters (Å, °)

**Table:**

Ni1—N1	1.880 (3)	C4—C5	1.522 (5)
Ni1—N3	1.888 (3)	C4—C6	1.538 (5)
Ni1—N2	1.916 (2)	C5—H5A	0.9600
Ni1—N4	1.917 (2)	C5—H5B	0.9600
Ni1—S1	3.2979 (13)	C5—H5C	0.9600
S1—C17	1.620 (5)	C6—H6A	0.9600
Cl1—O2'	1.359 (18)	C6—H6B	0.9600
Cl1—O1	1.369 (4)	C6—H6C	0.9600
Cl1—O3	1.385 (18)	C7—C8	1.494 (5)
Cl1—O2	1.40 (3)	C7—H7A	0.9700
Cl1—O4'	1.40 (4)	C7—H7B	0.9700
Cl1—O4	1.408 (11)	C8—H8A	0.9700
Cl1—O3'	1.41 (3)	C8—H8B	0.9700
N1—C2	1.284 (4)	C9—C10	1.505 (5)
N1—C16	1.482 (4)	C9—H9A	0.9600
N2—C7	1.487 (4)	C9—H9B	0.9600
N2—C4	1.505 (4)	C9—H9C	0.9600
N2—H2	0.9100	C10—C11	1.495 (5)
N3—C10	1.278 (4)	C11—C12	1.529 (4)
N3—C8	1.482 (4)	C11—H11A	0.9700
N4—C15	1.489 (4)	C11—H11B	0.9700
N4—C12	1.508 (4)	C12—C13	1.526 (4)
N4—H4	0.9100	C12—C14	1.531 (4)
N5—C17	1.159 (5)	C13—H13A	0.9600
O5—H5F	0.8500	C13—H13B	0.9600
O5—H5G	0.8500	C13—H13C	0.9600
C1—C2	1.491 (5)	C14—H14A	0.9600
C1—H1A	0.9600	C14—H14B	0.9600
C1—H1B	0.9600	C14—H14C	0.9600
C1—H1C	0.9600	C15—C16	1.498 (5)
C2—C3	1.496 (5)	C15—H15A	0.9700
C3—C4	1.523 (5)	C15—H15B	0.9700
C3—H3A	0.9700	C16—H16A	0.9700
C3—H3B	0.9700	C16—H16B	0.9700
			
N1—Ni1—N3	174.53 (11)	C3—C4—C6	107.2 (3)
N1—Ni1—N2	92.60 (11)	C4—C5—H5A	109.5
N3—Ni1—N2	88.02 (11)	C4—C5—H5B	109.5
N1—Ni1—N4	87.93 (11)	H5A—C5—H5B	109.5
N3—Ni1—N4	91.75 (11)	C4—C5—H5C	109.5
N2—Ni1—N4	176.80 (11)	H5A—C5—H5C	109.5
N1—Ni1—S1	92.86 (8)	H5B—C5—H5C	109.5
N3—Ni1—S1	81.67 (8)	C4—C6—H6A	109.5
N2—Ni1—S1	92.88 (8)	C4—C6—H6B	109.5
N4—Ni1—S1	90.25 (8)	H6A—C6—H6B	109.5
C17—S1—Ni1	85.87 (14)	C4—C6—H6C	109.5
O2'—Cl1—O1	127.9 (17)	H6A—C6—H6C	109.5
O2'—Cl1—O3	75.5 (14)	H6B—C6—H6C	109.5
O1—Cl1—O3	115.2 (10)	N2—C7—C8	108.2 (3)
O2'—Cl1—O2	113.7 (19)	N2—C7—H7A	110.0
O1—Cl1—O2	110.6 (14)	C8—C7—H7A	110.0
O3—Cl1—O2	107.7 (14)	N2—C7—H7B	110.0
O2'—Cl1—O4'	99 (2)	C8—C7—H7B	110.0
O1—Cl1—O4'	115 (2)	H7A—C7—H7B	108.4
O3—Cl1—O4'	119 (2)	N3—C8—C7	105.6 (3)
O2—Cl1—O4'	21 (2)	N3—C8—H8A	110.6
O2'—Cl1—O4	38.1 (9)	C7—C8—H8A	110.6
O1—Cl1—O4	97.2 (13)	N3—C8—H8B	110.6
O3—Cl1—O4	109.6 (11)	C7—C8—H8B	110.6
O2—Cl1—O4	116.7 (11)	H8A—C8—H8B	108.7
O4'—Cl1—O4	96.2 (18)	C10—C9—H9A	109.5
O2'—Cl1—O3'	103.0 (12)	C10—C9—H9B	109.5
O1—Cl1—O3'	99.4 (17)	H9A—C9—H9B	109.5
O3—Cl1—O3'	27.7 (17)	C10—C9—H9C	109.5
O2—Cl1—O3'	94.5 (16)	H9A—C9—H9C	109.5
O4'—Cl1—O3'	113 (2)	H9B—C9—H9C	109.5
O4—Cl1—O3'	136.4 (14)	N3—C10—C11	122.0 (3)
C2—N1—C16	120.5 (3)	N3—C10—C9	123.9 (3)
C2—N1—Ni1	130.8 (2)	C11—C10—C9	114.1 (3)
C16—N1—Ni1	108.6 (2)	C10—C11—C12	119.3 (3)
C7—N2—C4	114.5 (3)	C10—C11—H11A	107.5
C7—N2—Ni1	107.9 (2)	C12—C11—H11A	107.5
C4—N2—Ni1	114.08 (19)	C10—C11—H11B	107.5
C7—N2—H2	106.6	C12—C11—H11B	107.5
C4—N2—H2	106.6	H11A—C11—H11B	107.0
Ni1—N2—H2	106.6	N4—C12—C13	109.9 (3)
C10—N3—C8	120.7 (3)	N4—C12—C11	106.1 (3)
C10—N3—Ni1	129.9 (2)	C13—C12—C11	111.4 (3)
C8—N3—Ni1	109.0 (2)	N4—C12—C14	111.4 (3)
C15—N4—C12	115.1 (2)	C13—C12—C14	109.8 (3)
C15—N4—Ni1	109.0 (2)	C11—C12—C14	108.3 (3)
C12—N4—Ni1	112.87 (18)	C12—C13—H13A	109.5
C15—N4—H4	106.4	C12—C13—H13B	109.5
C12—N4—H4	106.4	H13A—C13—H13B	109.5
Ni1—N4—H4	106.4	C12—C13—H13C	109.5
H5F—O5—H5G	108.3	H13A—C13—H13C	109.5
C2—C1—H1A	109.5	H13B—C13—H13C	109.5
C2—C1—H1B	109.5	C12—C14—H14A	109.5
H1A—C1—H1B	109.5	C12—C14—H14B	109.5
C2—C1—H1C	109.5	H14A—C14—H14B	109.5
H1A—C1—H1C	109.5	C12—C14—H14C	109.5
H1B—C1—H1C	109.5	H14A—C14—H14C	109.5
N1—C2—C1	124.5 (3)	H14B—C14—H14C	109.5
N1—C2—C3	121.1 (3)	N4—C15—C16	107.5 (3)
C1—C2—C3	114.4 (3)	N4—C15—H15A	110.2
C2—C3—C4	120.4 (3)	C16—C15—H15A	110.2
C2—C3—H3A	107.2	N4—C15—H15B	110.2
C4—C3—H3A	107.2	C16—C15—H15B	110.2
C2—C3—H3B	107.2	H15A—C15—H15B	108.5
C4—C3—H3B	107.2	N1—C16—C15	106.6 (3)
H3A—C3—H3B	106.9	N1—C16—H16A	110.4
N2—C4—C5	110.3 (3)	C15—C16—H16A	110.4
N2—C4—C3	107.6 (3)	N1—C16—H16B	110.4
C5—C4—C3	110.8 (3)	C15—C16—H16B	110.4
N2—C4—C6	110.3 (3)	H16A—C16—H16B	108.6
C5—C4—C6	110.7 (3)	N5—C17—S1	176.7 (4)
			
N1—Ni1—S1—C17	−106.07 (18)	Ni1—N1—C2—C3	−0.1 (5)
N3—Ni1—S1—C17	73.60 (18)	N1—C2—C3—C4	−16.4 (5)
N2—Ni1—S1—C17	161.18 (18)	C1—C2—C3—C4	164.7 (3)
N4—Ni1—S1—C17	−18.13 (18)	C7—N2—C4—C5	−72.0 (3)
N3—Ni1—N1—C2	−110.1 (12)	Ni1—N2—C4—C5	53.0 (3)
N2—Ni1—N1—C2	−13.6 (3)	C7—N2—C4—C3	167.1 (3)
N4—Ni1—N1—C2	163.2 (3)	Ni1—N2—C4—C3	−67.9 (3)
S1—Ni1—N1—C2	−106.7 (3)	C7—N2—C4—C6	50.5 (4)
N3—Ni1—N1—C16	66.7 (12)	Ni1—N2—C4—C6	175.5 (2)
N2—Ni1—N1—C16	163.1 (2)	C2—C3—C4—N2	50.8 (4)
N4—Ni1—N1—C16	−20.1 (2)	C2—C3—C4—C5	−69.8 (4)
S1—Ni1—N1—C16	70.1 (2)	C2—C3—C4—C6	169.4 (3)
N1—Ni1—N2—C7	175.6 (2)	C4—N2—C7—C8	163.6 (3)
N3—Ni1—N2—C7	−9.8 (2)	Ni1—N2—C7—C8	35.4 (3)
N4—Ni1—N2—C7	76 (2)	C10—N3—C8—C7	−145.6 (3)
S1—Ni1—N2—C7	−91.4 (2)	Ni1—N3—C8—C7	41.0 (3)
N1—Ni1—N2—C4	47.2 (2)	N2—C7—C8—N3	−49.6 (4)
N3—Ni1—N2—C4	−138.3 (2)	C8—N3—C10—C11	176.2 (3)
N4—Ni1—N2—C4	−52 (2)	Ni1—N3—C10—C11	−12.0 (5)
S1—Ni1—N2—C4	140.2 (2)	C8—N3—C10—C9	−4.5 (5)
N1—Ni1—N3—C10	−93.8 (12)	Ni1—N3—C10—C9	167.3 (3)
N2—Ni1—N3—C10	169.6 (3)	N3—C10—C11—C12	−8.1 (5)
N4—Ni1—N3—C10	−7.2 (3)	C9—C10—C11—C12	172.5 (3)
S1—Ni1—N3—C10	−97.2 (3)	C15—N4—C12—C13	−78.0 (3)
N1—Ni1—N3—C8	78.7 (12)	Ni1—N4—C12—C13	48.0 (3)
N2—Ni1—N3—C8	−17.9 (2)	C15—N4—C12—C11	161.5 (3)
N4—Ni1—N3—C8	165.3 (2)	Ni1—N4—C12—C11	−72.5 (3)
S1—Ni1—N3—C8	75.3 (2)	C15—N4—C12—C14	43.8 (4)
N1—Ni1—N4—C15	−7.0 (2)	Ni1—N4—C12—C14	169.8 (2)
N3—Ni1—N4—C15	178.5 (2)	C10—C11—C12—N4	50.3 (4)
N2—Ni1—N4—C15	92.6 (19)	C10—C11—C12—C13	−69.3 (4)
S1—Ni1—N4—C15	−99.9 (2)	C10—C11—C12—C14	169.9 (3)
N1—Ni1—N4—C12	−136.2 (2)	C12—N4—C15—C16	160.1 (3)
N3—Ni1—N4—C12	49.2 (2)	Ni1—N4—C15—C16	32.1 (3)
N2—Ni1—N4—C12	−37 (2)	C2—N1—C16—C15	−140.5 (3)
S1—Ni1—N4—C12	130.90 (19)	Ni1—N1—C16—C15	42.3 (3)
C16—N1—C2—C1	2.4 (5)	N4—C15—C16—N1	−48.1 (4)
Ni1—N1—C2—C1	178.8 (2)	Ni1—S1—C17—N5	31 (8)
C16—N1—C2—C3	−176.5 (3)		

### Hydrogen-bond geometry (Å, °)

**Table:**

*D*—H···*A*	*D*—H	H···*A*	*D*···*A*	*D*—H···*A*
N2—H2···O2	0.91	2.28	3.16 (3)	162
N4—H4···N5	0.91	2.33	3.241 (5)	175
O5—H5F···N5	0.85	2.09	2.942 (6)	178
O5—H5G···N5^i^	0.85	2.15	2.997 (6)	178
C3—H3A···O4^ii^	0.97	2.49	3.450 (16)	172
C3—H3B···O2	0.97	2.48	3.26 (3)	137
C15—H15A···O1^iii^	0.97	2.37	3.155 (6)	138

Symmetry codes:  (i) −*x*+1, −*y*+2, −*z*+1; (ii) −*x*+1, −*y*+1, −*z*+2; (iii) *x*−1, *y*+1, *z*.
